# Developing a National Newborn Genomes Program: An Approach Driven by Ethics, Engagement and Co-design

**DOI:** 10.3389/fgene.2022.866168

**Published:** 2022-05-30

**Authors:** Amanda Pichini, Arzoo Ahmed, Christine Patch, David Bick, Mathilde Leblond, Dalia Kasperaviciute, Dasha Deen, Simon Wilde, Sofia Garcia Noriega, Christella Matoko, Alice Tuff-Lacey, Chris Wigley, Richard H. Scott

**Affiliations:** ^1^ Genomics England, London, United Kingdom; ^2^ Engagement and Society, Wellcome Connecting Science, Wellcome Genome Campus, Hinxton, United Kingdom

**Keywords:** newborn screening, whole genome sequencing, rare diseases, public health, ethics, public engagement, co-design

## Abstract

The transformative potential of whole genome sequencing (WGS) as a diagnostic tool in healthcare has been demonstrated by initiatives including the 100,000 Genomes Project and is now offered to certain patients in the National Health Service (NHS) in England. Building on these foundations, the utility of WGS in the newborn period can now be explored. Genomics England is working in partnership with NHS England and NHS Improvement and other healthcare, patient and public interest groups to design a research program embedded in the NHS to explore the potential challenges and implications of offering WGS in all newborns. The program will aim to: 1) evaluate the feasibility, utility and impact on the NHS of screening for childhood-onset rare actionable genetic conditions; 2) understand how, with consent, genomic and healthcare data could be used to enable research to develop new diagnostics and treatments; and 3) explore the implications of storing an individual’s genome for use over their lifetime. Recognizing the important practical, scientific and ethical questions that we must explore in dialogue with the public and experts, we are taking a collaborative, evidence-based and ethically deliberate approach to designing the program. An iterative co-design process including a nationwide public dialogue has identified emergent themes and ethical considerations which are the focus of the program’s design. These themes will be further developed through continued engagement with healthcare professionals, researchers, ethics experts, patient groups and the public, with an ongoing commitment to embedding ongoing ethics research and co-design into the delivery of the program.

## Introduction

The United Kingdom (UK) has consistently taken the lead to introduce genomic technologies into healthcare and research, particularly whole genome sequencing (WGS). Initiatives such as the 100,000 Genomes Project and the National Health Service (NHS) Genomic Medicine Service in England have demonstrated the potential of WGS to increase the diagnostic yield for a range of rare conditions and its role in cancer (Turnbull et al., 2018; Smedley et al., 2021). In the UK, newborn screening is provided by the NHS on the basis of recommendations from the UK National Screening Committee and consists of a physical examination, hearing screen and a blood spot test. The blood spot test directly screens for nine rare conditions, for which there is substantial evidence that early identification and treatment can improve health outcomes (NHS, 2022). Parental consent is required, and there is high uptake with 95–99% of newborns screened (GOV.UK, 2022). The UK tests for fewer conditions than other high-income countries, and there is growing recognition of the potential of early and pre-symptomatic detection of a larger number of conditions to provide benefits to the child and their family, particularly highlighted by rare disease communities. This may be done through the expansion of genomic and/or other technologies, and by reviewing the evidence required to incorporate conditions in screening programs in the context of a national publicly-funded health system (Genetic Alliance UK, 2022). Other genomic population screening research initiatives have taken place or are underway internationally, and highlight the importance of equitable access, managing expectations and uncertainties, and ensuring a robust consent process (Screen4care, 2022; Holm et al., 2018; Roman et al., 2020; Downie et al., 2021). However, there remains a relative lack of empirical evidence about the benefits and harms of these programs, particularly in the long term.

The UK Chief Medical Officer emphasized the importance of providing expanded and equitable access to genomic services in her 2016 Annual Report and requested a group to investigate the benefits of genomic analysis in children including in the context of newborn screening (Department of Health and Social Care, 2017). The Genomic Analysis in Children Task and Finish Group—made up of experts from laboratory and clinical genomics, ethics and screening as well as patient and parent representatives—highlighted that WGS has the potential to add to current aspects of the newborn screening program, as well as provide additional opportunities for ongoing research and feedback of information beyond the newborn period. An initial conservative analysis of rare inherited conditions suggests that 1 in 260 live births are affected with a condition for which identification through WGS has the potential to reduce or avoid harm in early life. The group recommended the initiation of a large scale, resourced research program in the UK to gather evidence on the effectiveness, feasibility and acceptability of WGS for screening in newborns (Genomics England, 2022a).

Genomics England is working in partnership with NHS England and NHS Improvement as well as a range of healthcare, patient and public interest groups to develop this program. A recently published vision outlines three distinct but related aims of the Newborn Genomes Program (Genomics England, 2022a):

1) to identify a larger number of rare and actionable conditions than currently screened for;
2) to enable research on genomic and health data from newborns to further develop diagnostics and treatments; and
3) to explore the potential benefits, risks and broader implications of storing an individual’s genome for use over their lifetime.

These aims will be explored through a research pilot aimed to start in 2023, guided by a protocol subject to research ethics approval, and crucially embedded within the NHS. This would include at least 100,000 babies, powered to provide the data required to determine the effectiveness of WGS in the newborn screening context based on modelling of likely incidence of conditions targeted and conservative estimates of sensitivity and specificity (Genomics England, 2022a). An NHS Steering Group has been established to provide advice and expertise around decisions being made about the design of the program, and ensure that any learnings can be effectively translated from research to clinical care in a nationwide health system.

WGS has increasingly demonstrated the ability to detect a broad range of genomic variants using a single technology, with costs, sequencing and analysis times decreasing to provide results where an intervention may be time-sensitive. This technology provides flexibility to analyze additional variants when new evidence about pathogenicity or treatment would support their inclusion in newborn screening, or to analyze in a diagnostic context if symptoms arise in an individual in the future, without requiring new or additional samples (Belkadi et al., 2015; Dimmock et al., 2021). WGS also provides great value for research discovery, with potential for genome-wide research to identify new diagnoses, diagnostics and treatments, and allows for a greater understanding of the relevance of particular genetic variants to health and disease. This could be supported using the successful model that Genomics England has developed in collaboration with its participants, where de-identified genomic and health data are presented in a trusted research environment to accredited researchers for agreed purposes with access controlled by participant-led governance.

Despite these advantages, the use of WGS in newborn screening at a national health system level is a novel approach and limitations remain, particularly when testing asymptomatic rather than pre-symptomatic individuals. For example, it will be important to minimize feedback of information that is uncertain or not clinically useful, and the burden this may place on families and health systems (Nuffield Council on Bioethics, 2017; Biesecker et al., 2021; Downie et al., 2021). The sensitivity, specificity, positive and negative predictive values would be expected to vary for each condition depending on its prevalence, ability to distinguish pathogenic from benign variants, and ability to detect known and unknown pathogenic variants (Hagenkord et al., 2020; Marshall et al., 2020). Changing any of these metrics could result in under or over-diagnosis of any of these conditions, or missing diagnoses. This necessitates careful thought to determine which conditions will be analyzed and fed back in the newborn period, requiring the establishment of clear pathways to additional investigations such as biochemical tests to confirm diagnoses or clarify any findings. Challenges also remain with regards to re-analysis of data over time, and how to manage initial and ongoing consent.

Taking into account different perspectives, the team are embracing a collaborative approach and ongoing commitment to openness, grounded in national dialogue and research with experts and the public. This paper will outline our approach to engagement, co-design and ethical considerations that are required to ensure a transparent and evidence-based program within a nationwide publicly funded health system.

### Public Dialogue and Engagement

Research in this area—just as for any population screening program that might follow—must be premised on public acceptance and support. This is not a one-off process but one of ongoing dialogue and adaptation as expectations emerge and evidence develops. In 2020-2021, a national dialogue commissioned by Genomics England, the UK National Screening Committee and United Kingdom Research and Innovation’s Sciencewise program, was carried out with members of the UK public (Van Mil, 2021). This was a novel approach to ensuring that the public’s views directly impacted the initiation and design of a nationwide population screening-based research program. 133 participants reflective of the UK population each took part in a series of interviews and group workshop sessions, which were recorded and analyzed using grounded theory methodology. Participants expressed broad support for the potential use of WGS for newborn screening, whilst also raising a number of issues and principles that would need to be addressed before this could be initiated in practice (Van Mil, 2021). Further engagement with stakeholders including patients and families with rare conditions, public interest groups, policy and commissioning services, ethics experts, healthcare professionals and Royal Colleges, laboratory and diagnostic services and researchers have echoed similar considerations (Genomics England, 2022a). These and the considerations raised in the public dialogue have been grouped into six emergent themes which will be discussed further in this paper and guide the program as it continues to develop:

1) The benefits, limitations, and unknowns of WGS as a screening tool;
2) Principles for including conditions in the screening panel, co-developed with relevant stakeholders;
3) Person-centered consent across screening, research and reanalysis;
4) A supportive and inclusive experience for all families;
5) Trusted and future-proofed genomic data storage and usage; and
6) A sustainable and scalable program for the NHS, should the evidence generated from the pilot support a future clinical service.

### Ethical Implications of Whole Genome Sequencing in Newborns

Alongside public dialogue and engagement, ethics will be central to the co-design of the program and an ongoing component of the research pilot itself.

The three aims of the Newborn Genomes Program each raise distinct, yet related, ethical considerations that will need to be explored prior to, throughout, and beyond, the duration of the program. Initial ethical themes which have been raised through the public dialogue and ongoing stakeholder engagement, reflecting previous research include (Botkin and Rothwell, 2016; Friedman et al., 2017; Nuffield Council on Bioethics, 2017; Sénécal et al., 2018; Goldenberg et al., 2019; Biesecker et al., 2021; Levy, 2021): consent, specifically considering the context of genomics in screening; the benefits and harms of results in a pre-symptomatic context (such as uncertainty, overmedicalization, genetic determinism, and the psycho-social impacts on parent-child relationships); data governance including storage, access and use by clinical, academic and life sciences industry partners including access requests by parents; balancing the rights and needs of the child with those of the wider family; equitable access and the potential for discrimination; resource utilization and prioritization; and broader societal implications and future unintended consequences. It will be important to identify whether there are novel ethical areas for consideration in the newborn context which will need to be included in the ethics agenda for the program.

Crucially, the program aims to incorporate ethics not only in the context of an underlying research-ethics approved protocol, but also as an inherent part of program by embedding ethics throughout the governance, design, implementation and evaluation. An initial set of foundational ethical principles and commitments are being developed and will evolve into an ethical framework including different positions for each of the three aims of the program, developed through a combination of ethics research, engagement and deliberation with experts and a diverse range of publics. Genomics England’s existing Ethics Advisory Committee, Participant Panel and internal Ethics team, a dedicated newborn ethics working group, as well as external stakeholder and public engagement activities including young people and expectant parents, will offer insights to ethical matters arising in relation to the program with a focus on ensuring ongoing trustworthiness. The program provides opportunities to test these ethical and social dimensions before, during and after the pilot, to broaden our insight and foresight for the program and any related future developments. Furthermore, the program intends to facilitate and inform broader ethical debates which stretch beyond the research pilot, particularly in relation to the possibilities and challenges of using the genome as a lifetime clinical resource.

### What Does it Mean to Co-design?

The principles of experience-based co-design underlie our approach to designing the program in an iterative manner (Donetto et al., 2015). In line with this approach, working groups are being developed with representation across the country from different stakeholders (including healthcare professionals, researchers, scientists, patients and members of the public) to provide advice and recommendations regarding the design of the program. Outputs from these groups would feed in to the NHS Steering Group and existing governance structures within Genomics England to inform delivery of the pilot. Here, we provide two illustrative examples.

In contrast to other state screening programs or related research programs where criteria are typically informed exclusively by clinicians, policymakers and researchers, we have included wider views of the public, rare disease patient communities and ethics experts, reflecting our focus on the importance of public acceptability of a nationwide research program. A working group of 28 individuals reflecting these various areas of expertise has been established to develop a set of principles using consensus methodology, which will inform the conditions (and the genes and variants that cause them) that could be initially analyzed, as well as an approach to an ongoing review process where conditions may be added or removed based on new information. While there are arguably many possible answers to this question, the overarching view from the public dialogue is being used as a starting point: to broadly focus on conditions that have an impact in early childhood, and where there are intervention(s) that can cure, prevent or slow progression. Consideration must also be afforded to conditions which would demonstrate cost-effectiveness for a publicly-funded national health service, and whether the condition has an established follow-on test(s) and care pathway across the NHS with identified specialists who could provide care and follow up support. Once the principles have been established by the working group, they will be applied to genes, followed by variant curation and rigorous empirical analysis to estimate the false positive and false negative rates of the variant detections in the selected genes. There are a number of processes that have been published to generate a list of genes that will be drawn upon (Ceyhan- Birsoy et al., 2017; Milko et al., 2019; Downie et al., 2021; Bick et al 2020). These principles and the final list of conditions, genes and variants will be made available for deliberative debate for further input from professional, patient and public groups across the UK.

Another working group is focusing on the recruitment process for parents who may consider participating in the pilot through consenting on behalf of their newborn. This group includes a range of healthcare professionals including midwives, as well as parent and patient representatives with a variety of perspectives. It is critical that the pilot will be understandable and desirable to parents of all backgrounds, to enable informed decision making about taking part as well as ensuring equity of access. As such, the group meet regularly to brainstorm and share their thoughts on the recruitment materials, messages, and the process of recruitment for the pilot. The concepts developed in these group sessions are then taken out and tested with healthcare professionals and expecting parents across the UK in an iterative learning process, including a focus on traditionally underserved groups in genomic research.

Additional working groups that have or will be initiated in the coming months will focus on education and training for the workforce; consent including parents’ initial decision to join the program as well as the need for young people to review their decision at 16 and the ongoing opportunity to withdraw; treatment and support pathways for families receiving results; sampling and sequencing approaches; and how the program will be evaluated.

## Discussion

The United Kingdom is uniquely positioned to build on the foundations of WGS in a diagnostic context and design a program to gather evidence on the effectiveness, feasibility and acceptability of WGS in newborns. This ambitious research program of genomics in newborn screening is the largest to date, with an opportunity to assess the benefits and challenges of this approach at an unprecedented scale and within a nationwide publicly funded health service. Furthermore, due to the NHS’s already close integration with genomic research, experience gained throughout the program could be more seamlessly translated into clinical practice in an equitable and cost-effective manner. This is in contrast to other newborn screening initiatives involving genomics which involve distinctly separate research pathways, and one or a small number of hospital systems (Downie et al 2021). Our proposed approach involves prioritizing nationwide engagement, co-design and ethical considerations to directly feed into decisions made about the program, and as key components to ensuring that the benefits, practicalities and challenges of this program can be realized. This focusses on a commitment to involving the public and patient communities in shared decision-making about programs that will impact on population health.

There are a number of implications that will be the focus of program design in the coming months, building on the challenges and learnings from the implementation of the 100,000 Genomes Project and other national screening initiatives. As a research program where results will be fed back *via* clinical pathways in a number of hospitals and community health services across the country, there is a need to consider the time, training and resource requirements from the point of recruitment through to ongoing care, with interactions needing to be carefully monitored to ensure that the research pilot is not affecting uptake of the current newborn screening program. Sampling, sequencing and bioinformatic pipelines, laboratories and reporting systems must be capable of processing samples at scale and in a time frame that can allow for treatment to be rapidly initiated, within days for some conditions. There must be a clear plan as well as adequate support and information available for those families where a rare condition is identified. To consider the potential of this as a future national clinical service, the program would not only need to demonstrate evidence of benefit and cost-effectiveness and the ability to maintain trust and high ethical standards, but also be operationally feasible at scale within a national publicly-funded health system. In order to effectively capture and assess outcomes of this program a co-designed robust evaluation framework will be devised to ensure that technology performance, health outcomes, implementation, psychosocial and ethical issues can be monitored. This will include both qualitative and quantitative metrics, and ensure that any evidence can be independently evaluated in a formative manner to be able to adapt and improve processes throughout the course of the pilot.

Factors influencing the adoption of WGS in newborn screening will likely reflect many of those already known to impact adoption of population-wide screening, genomic testing and other novel technologies, and will be explored throughout the course of this program alongside other emergent issues (Dheensa et al., 2019; Best et al., 2021; Sanderson et al., 2022). At a more individual level, factors include perceived relevance to one’s own health or their family; prior experiences with screening and health care; time and resources available to access and understand information to make an informed choice; engagement and leadership from trusted sources; as well as cultural, religious, familial and personal values. Factors at a health systems level include organizational culture and leadership, perceived relevance to one’s clinical practice, access to education and training, and ability and capacity to work with colleagues within and across specialties to make complex pathways work seamlessly. At a broader societal level, public acceptability and trustworthy systems and organizations are imperative, particularly in the context of population-wide screening in a publicly-funded national health system. Crucially, the ethically-focused and collaborative aspects of the design and development of the Newborn Genomes Program are expected to continue throughout the duration of the pilot, reflecting a commitment to transparency, trustworthiness and learning at every step.

## Data Availability

The original contributions presented in the study are included in the article, further inquiries can be directed to the corresponding author.
